# Supplementary material for: Co-Adsorption of H2O, OH, and Cl on Aluminum and Intermetallic Surfaces and Its Effects on the Work Function Studied by DFT Calculations
Source: Molecules. 2019 Nov 25;24(23):4284. doi: 10.3390/molecules24234284 (PMC6930550; doi:10.3390/molecules24234284)
Supplement: Supplementary file 1 [file molecules-24-04284-s001.pdf]

Supplementary Information

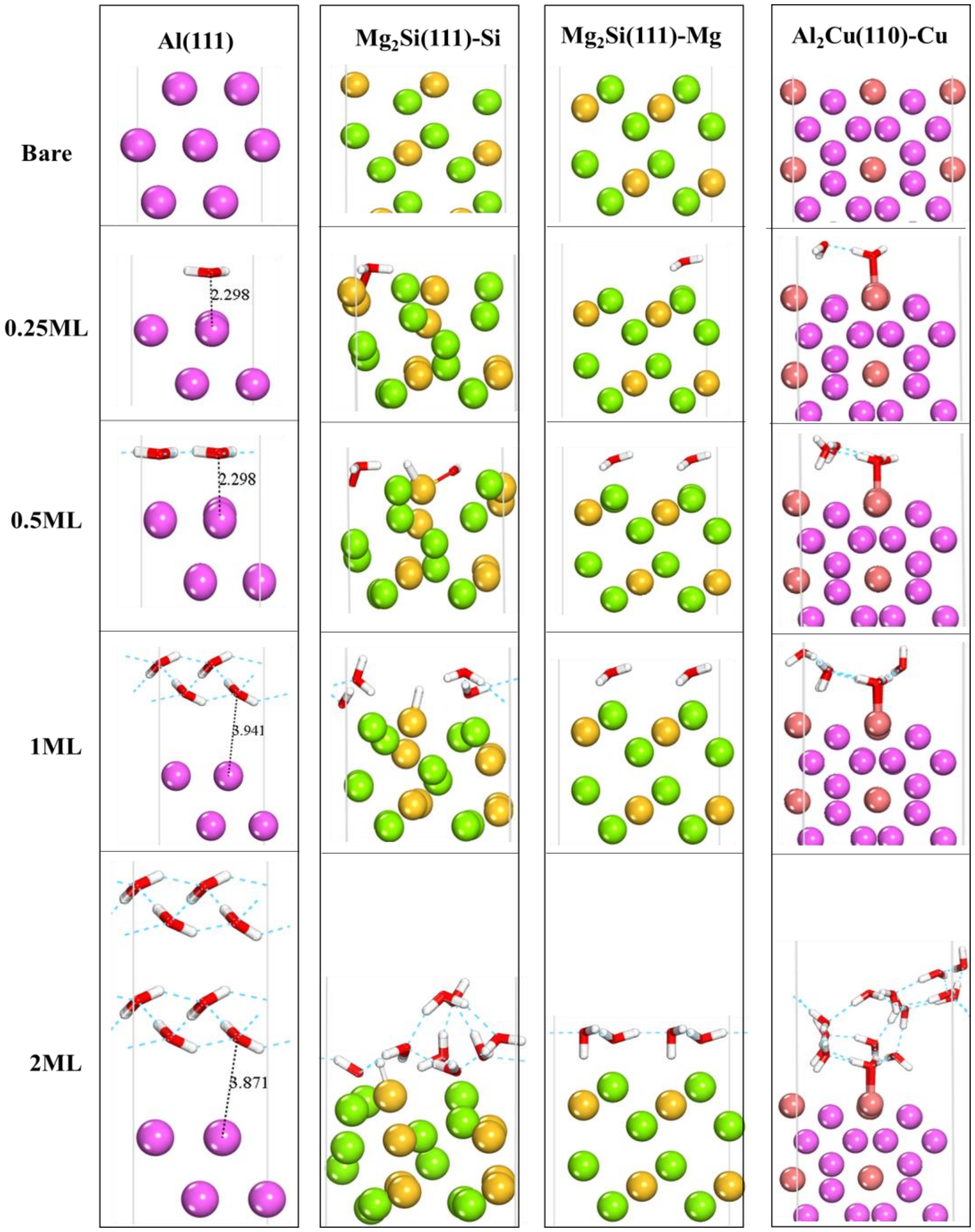

Figure S1. Optimized structures of pure H_2O on Al(111), Mg_2Si(111)–Si, Mg_2Si(111)–Mg and Al_2Cu(110)–Cu surfaces with increasing H_2O coverage. The structures are presented as side views. Colors: Al magenta, O red, H white, Si yellow, Mg green, Cu orange.

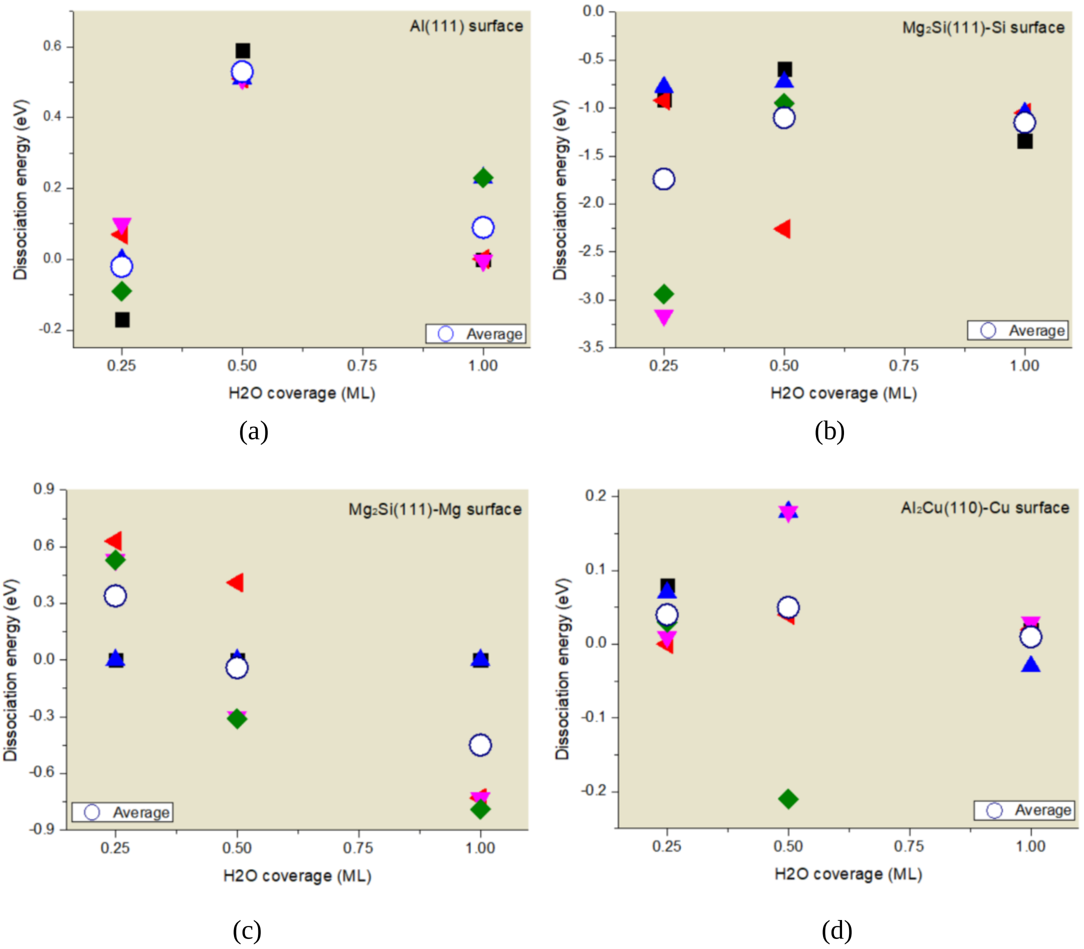

Figure S2. The dissociation energies (E_d, solid points) of one H_2O corresponding to different dissociation paths on (**a**) Al(111), (**b**) Mg_2Si(111)–Si, (**c**) Mg_2Si(111)–Mg, (**d**) Al_2Cu(110)–Cu surfaces plotted against H_2O coverage. Average $\overline{E_d}$ is displayed with hollow points in each figure.

Table S1. Minimum H_2O dissociation energy ($E_{d\ min}$) and substitution energy (E_s) corresponding to the substitution of OH with Cl at increasing H_2O coverage.

H_2O coverage / Surfaces	$E_{d\ min}$/eV			E_s/eV		
	0.25ML	0.5ML	1ML	0.25ML	0.5ML	1ML
Al	-0.12	0.51	-0.004	0.56	**-0.19**	0.98
$Mg_2Si(111)$–Si	-3.16	-2.26	-1.34	**-0.05**	**-0.11**	0.29
$Mg_2Si(111)$–Mg	~0	-0.31	-0.79	0.50	0.33	0.37
$Al_2Cu(110)$–Cu	~0	-0.21	-0.03	1.21	0.21	0.10

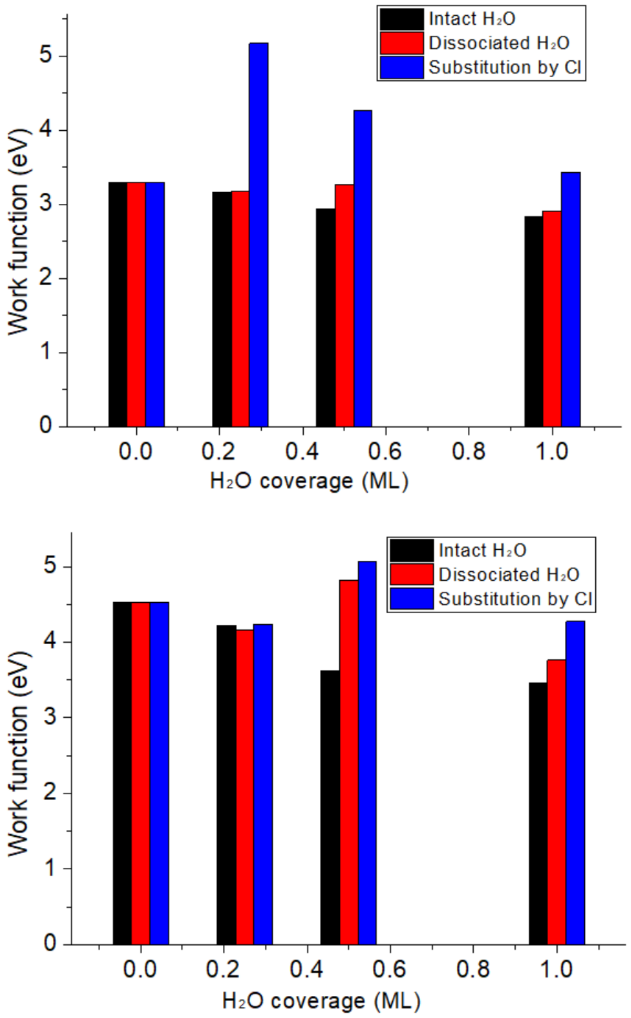

Figure S3. Work function change on Mg_2Si(111)–Mg (above) and Al_2Cu(110)–Cu surfaces (bottom), with INT, DIS, SUB aqueous ad-layer. Data for bare surfaces and INT aqueous ad-layer systems are from our earlier work[10,33].

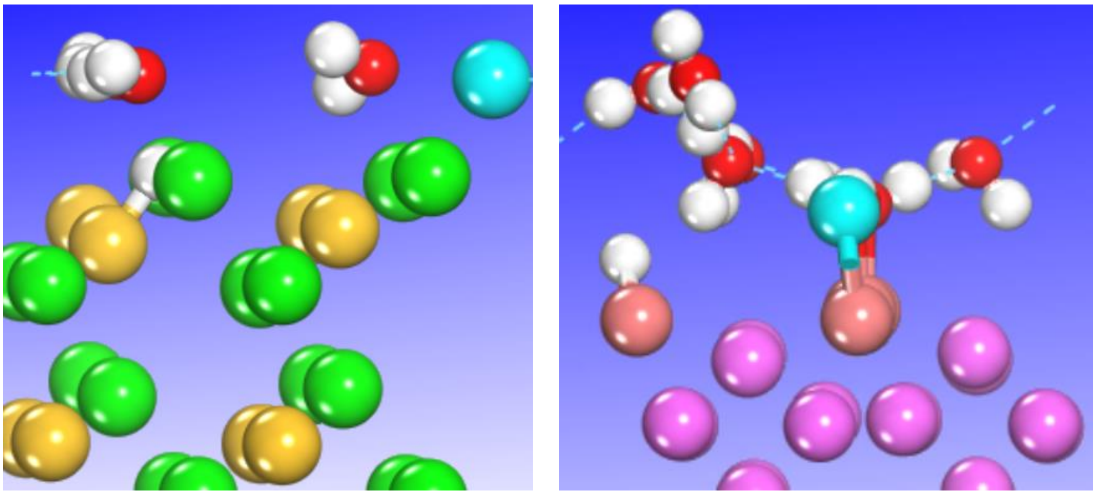

Figure S4. Optimized structure of Cl-containing aqueous ad-layer on Mg_2Si–Si (left) and Al_2Cu–Cu (right). Refer to Figure S1 for atomic colors.
